# Global transcription regulation of RK2 plasmids: a case study in the combined use of dynamical mathematical models and statistical inference for integration of experimental data and hypothesis exploration

**DOI:** 10.1186/1752-0509-5-119

**Published:** 2011-07-29

**Authors:** Dorota Herman, Christopher M Thomas, Dov J Stekel

**Affiliations:** 1 Center for Systems Biology, School of Biosciences, University of Birmingham, Edgbaston, Birmingham B15 2TT, UK; 2 School of Biosciences, University of Birmingham, Edgbaston, Birmingham B15 2TT, UK; 3 Integrative Systems Biology, School of Biosciences, University of Nottingham, LE12 5RD, UK

## Abstract

**Background:**

IncP-1 plasmids are broad host range plasmids that have been found in clinical and environmental bacteria. They often carry genes for antibiotic resistance or catabolic pathways. The archetypal IncP-1 plasmid RK2 is a well-characterized biological system, with a fully sequenced and annotated genome and wide range of experimental measurements. Its central control operon, encoding two global regulators KorA and KorB, is a natural example of a negatively self-regulated operon. To increase our understanding of the regulation of this operon, we have constructed a dynamical mathematical model using Ordinary Differential Equations, and employed a Bayesian inference scheme, Markov Chain Monte Carlo (MCMC) using the Metropolis-Hastings algorithm, as a way of integrating experimental measurements and a priori knowledge. We also compared MCMC and Metabolic Control Analysis (MCA) as approaches for determining the sensitivity of model parameters.

**Results:**

We identified two distinct sets of parameter values, with different biological interpretations, that fit and explain the experimental data. This allowed us to highlight the proportion of repressor protein as dimers as a key experimental measurement defining the dynamics of the system. Analysis of joint posterior distributions led to the identification of correlations between parameters for protein synthesis and partial repression by KorA or KorB dimers, indicating the necessary use of joint posteriors for correct parameter estimation. Using MCA, we demonstrated that the system is highly sensitive to the growth rate but insensitive to repressor monomerization rates in their selected value regions; the latter outcome was also confirmed by MCMC. Finally, by examining a series of different model refinements for partial repression by KorA or KorB dimers alone, we showed that a model including partial repression by KorA and KorB was most compatible with existing experimental data.

**Conclusions:**

We have demonstrated that the combination of dynamical mathematical models with Bayesian inference is valuable in integrating diverse experimental data and identifying key determinants and parameters for the IncP-1 central control operon. Moreover, we have shown that Bayesian inference and MCA are complementary methods for identification of sensitive parameters. We propose that this demonstrates generic value in applying this combination of approaches to systems biology dynamical modelling.

## Background

### IncP-1 Plasmids

Plasmids are autonomous, extra-chromosomal, self-replicating DNA elements typically associated with bacteria [1]. They are important as they can maintain and transfer genes for antibiotic resistance [2] and other important phenotypes, often as part of transposable elements [3]. They are also widely used as tools for genetic manipulation [4,5]. Finally, they serve as tractable models for a replicating chromosome and for evolution and co-evolution studies [6,7].

The low-copy number RK2 plasmid belongs to the plasmid incompatibility group P (IncP) of *Escherichia coli *(IncP-1 of Pseudomonas species). It can persist in most Gram-negative bacteria [8,9], and is thus referred to as having a broad host range. Moreover, RK2 plasmids can transfer conjugatively to Gram-positive bacteria [10] as well as to some eukaryotic cells [11] and yeasts [12]. RK2 and identical plasmids were first isolated from carbenicillin resistant *Pseudomonas aeruginosa *and *Klebsiella aerogenes *in 1969 [13]. Their complete sequence was first compiled in 1994 [13], and improved genome sequence published in 2007 [14].

**Figure 1:**
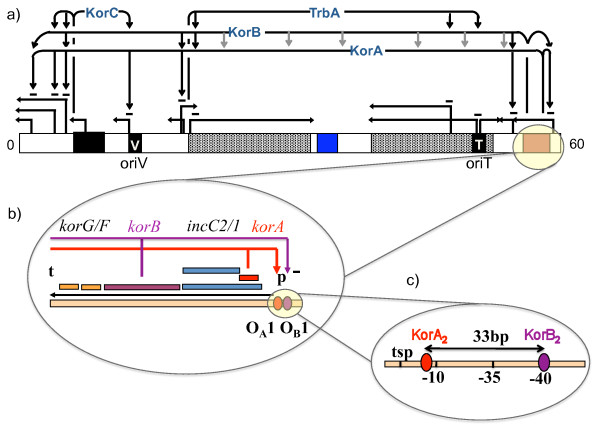
**Genome map of RK2 plasmid**. a) RK2 whole genome map, 60,099 bp; KorA, KorB, TrbA, KorC are global regulators. Arrows to the promoters and signs indicate the binding sites of the regulators and a type of regulation (- is repression), oriV and oriT are origins of vegetative replication and transfer, respectively, blac - Tn1 (transportable element), grey - Tra1 and Tra2 (transfer genes), blue - partitioning function, red - the central control operon. b) The central control operon, consisting of *korA, incC2/C1*, *korB*, *korG/F *genes, is regulated by a single promoter (p) and ends at a terminator (t). The operon is negatively and co-operatively auto-regulated by KorA and KorB. c) Binding sites of KorA and KorB on the *korAp *promoter: KorB binds 40 bp upstream of the transcription start point (tsp), and KorA binds 33 bp downstream from KorB binding site (O_B_1). KorA binds in -10 region, where RNAP binds.

The plasmid contains multiple genes encoding resistances to antibiotics including tetracycline and kanamycin as well as beta-lactam antibiotics. It is also able to accept and spread other transposable elements [15]. The genome is 60,099 bp (Figure 1), consisting of at least 74 genes and multiple regulatory circuits controlled by 7 transcriptional regulators, including four global regulators KorA, KorB, KorC and TrbA (Figure 1) [13]. KorA, which binds to the DNA strand as a dimer at seven sites (Figure 1), coordinates expression of genes for replication and stable inheritance [16,17]. KorA binding sites are located approximately 10 bp upstream of the transcription start points [13] and vary in terms of their affinity for the DNA strand that range between 12 and 272 nM [18]. KorB also binds to the DNA strand as a dimer (Figure 1), and is directly involved in partitioning, replication and stable maintenance [19-21]. Twelve KorB binding sites have been identified, with affinities ranging between 5 and 34 nM [22], and with proximity to binding sites for either KorA or TrbA [23,24]. The distances from operon transcription start points have been found to range from 39 to 2000 bp [25].

The RK2 plasmid genome encodes five promoters that are known or are predicted to be cooperatively regulated by KorA and KorB dimers (*korA*p, *kfrA*p, *trfA*p, *kleA*p, *klaA*p). Experiments have shown at least 3.4-fold cooperativity at *korA*p [24]. Therefore, cooperative binding of KorA and KorB to the DNA strand enhances the repression of genes from a particular operon. The most salient example of an operon regulated by KorA and KorB dimers on a single promoter, *korA*p, is the *korABF *operon itself, which encodes the two global regulators KorA and KorB, and is known as the central control operon (*cco*) [13]. Thus the *cco *is co-operatively and negatively autoregulated by KorA and KorB dimers. KorA dimers bind in the -10 region of *korA*p [18], overlapping the region where RNA polymerase (RNAP) binds to initiate transcription. KorB dimers bind 39/40 bp upstream of the transcription starting site, immediately upstream of the -35 region of the RNAP binding site; it is plausible that KorB allows RNAP to bind to the DNA strand but prevents it from opening the helix [26]. Nuclear Magnetic Resonance spectroscopy (NMR) and mutational analysis experiments have identified that tyrosine 84 on KorA is crucial for cooperativity between KorA and KorB [27]. Moreover, this residue is directly involved in the protein-protein interaction and has no impact on repression activity. Although the KorB dimer has two contact sites for KorA, it is currently thought that cooperating KorA and KorB act on the DNA strand as a complex in proportion 1:1 [27].

### Quantitative Experimental Measurements of the central control operon

Gene expression from the *cco *has been broadly examined experimentally. The abundances of KorA and KorB in exponential growth in *E.coli *have been measured in numbers of total monomers as 4000 (1600 nM) and 1000 (400 nM) respectively [18,28], and the abundance of KorB in a mutant, where co-operativity between KorA and KorB was knocked out by modification of tyrosine 84, was measured as 2300 monomers (920 nM) (unpublished data from Thomas *et al*.). Affinities of KorA and KorB to their binding sites at the promoter *korA*p have also been measured [18,22] and are 12.9 nM and 9.3 nM, respectively. Although affinities of KorA to the KorB-DNA complex and KorB to the KorA-DNA complex have not been measured, the affinity of KorB to the comparable IncC1-DNA complex has been measured as 3.1 nM [22]. Interestingly, the abundances of KorA and KorB proteins are more than two orders of magnitude higher than the affinities of these proteins to the auto-regulating binding sites.

There are varied data on the repression level of the *korA*p promoter. When KorA and KorB have been over-expressed relative to the wild-type, near total repression (> 800-fold) of the *cco *has been reported [24]. This indicates complete repression when both KorA and KorB dimers are bound to the DNA. A stronger signal of reporter protein expression has been demonstrated when either KorA or KorB dimers are bound alone. We refer to this phemonenon as partial repression. Recent quantitative work, where KorA and KorB expression was controlled on an inducible promoter at a similar copy number as in the wild type plasmid, suggests that the reduction in promoter activity by KorA and KorB at physiological levels is about 91.8-fold when compared to the case when repressors are absent [28].

The physical mechanisms for partial repression are unclear. Weaker reporter gene signals might result from a mixture of DNA molecules that are either repressor free and therefore "on" or occupied with repressor and therefore "off". Alternatively it may be that occupancy of the promoter by just one or other of the repressors bound to its operator site might just reduce the chance of promoter activity rather than complete inhibition. The difficulty of experimental investigation of these mechanisms arises because KorA or KorB alone would only be bound to the DNA for a small proportion of time.

### Mathematical Modelling Approaches

In this work we use mathematical models to better articulate our understanding of regulation of the *cco *by KorA and KorB. The models facilitate integration of the experimental data in the context of a formal description of the molecular processes of *cco *regulation. For the model itself, the approach we take is to derive a set of ordinary differential equations (ODEs) describing the dynamical processes that give rise to the measured protein concentrations, and thus to obtain a mathematical description of a biological mechanisms of interest - namely transcription regulation and protein synthesis.

Importantly, some of the available experimental data, such as affinities of binding sites, refer to dynamical processes of gene regulation, which can be thought of as parameters of a dynamical model. Other data, such as measurements of protein expression, are phenotypic, and can be thought of as outputs of a dynamical model. Some information, e.g. the rates of protein synthesis, is experimentally unavailable, and need to be estimated from the data that is available. This situation motivates the use of Bayesian inference [29] to integrate experimental measurements with the model and infer unknown parameters. Bayesian inference provides a robust probabilistic framework for capturing different sources of uncertainty in a modelling environment. It provides outputs in the form of posterior distributions for the parameters, that describe how likely it is that each of the parameters takes particular values, given the experimental measurements and model structure. The Bayesian framework has been recently used in systems biology [30] for different models including dynamical models described by ODEs [31] and stochastic models [32]. These uses include parameter inference for dynamical models of metabolic pathways using steady state measurements [33], for a dynamical model of gene regulation where time series measurements were used [34], or stochastic models of gene expression in single cells [32]. In this work, we employ a Bayesian Monte Carlo Markov Chain (MCMC) method known as the Metropolis-Hastings algorithm [35].

The posterior distributions of parameters obtained from Bayesian inference can be used to determine how sensitive a model is to that parameter: where the distribution is broad, then the model is less sensitive to that parameter, and where it is narrow it is more sensitive to that parameter. If when a prior distribution is supplied, for example for a measured parameter, the posterior distribution is similar to the prior distribution then no further information has been gained. However, if the posterior distribution is different, then additional information has been obtained.

A conceptually different approach to determining the sensitivity of a biological system model to its parameters is provided by Metabolic Control Analysis (MCA; [36,37]). This measures the extent to which an outcome of the model is sensitive to changes in parameter values. It gives sensitivity read-outs, referred to as control coefficients, based on analytic properties of a set of differential equations. These two approaches have been developed by distinct research communities and have rarely been applied for analysis of the same system.

### Aims of this study

In this study, we combine dynamical modelling, Bayesian inference and MCA to integrate the experimental data with our mechanistic knowledge of *cco *regulation, with the aim of addressing five specific research questions. First, are the measured data compatible with our understanding of the key mechanisms underlying the system operation? Specifically, can the cooperative model explain the high abundance of KorA and KorB proteins relative to their binding strengths? Second, is there sufficient information to robustly estimate unknown and unmeasured parameters, in particular the rates of protein synthesis and the rates at which KorA and KorB dimers separate into monomers, from the available data and molecular mechanisms. If not, what additional information is required? Third, to which parameters is the system sensitive, and to what degree? This knowledge will act as a guide for future experiments to determine what parameters to measure. Fourth, can we use the information about de-repression in the absence of KorA and KorB dimers to learn more about partial repression by these proteins, and thus refine the model? Fifth and finally, to what extent do Bayesian inference and MCA provide comparable or complementary information about parameter sensitivities of the same system of equations fitted to experimental data?

## Results

### Mathematical model for the regulation of KorA and KorB

We derived the model by first writing a chemical reaction scheme for the biological mechanisms; as this is not used further, the scheme is supplied as an additional file (see Additional file 1: Chemical reaction scheme). The model consists of equations for KorA monomers (*A*_1_), KorA dimers (*A*_2_), KorB monomers (*B*_1_) and KorB dimers (*B*_2_). The processes and parameters included in the model are: (i) associations and dissociations of KorA and KorB proteins to/from the DNA strand; (ii) their binding cooperativity; (iii) expression of KorA (*k*_A_) and KorB (*k*_B_) from the empty DNA strand (*D*); (iv) from the DNA strand bound by either KorA (*X*) or KorB (*Y*) dimers; (v) dimerizations (*λ*_A_, *λ*_B_) and monomerizations (*σ*_A_, *σ*_B_) of the KorA and KorB proteins; (vi) degradations/dilutions of all species (*γ*_P_). The equations are:(1)(2)(3)(4)

Equation 1 describes the rate of change of KorA monomers as a function of time. Protein synthesis is considered as a consolidated process that includes transcription, translation and mRNA turnover. This choice of abstraction is appropriate because we have experimental data on protein abundance and dilution, but no available data on mRNA abundance or turnover. The first term (*Dk*_A_) represents protein synthesis from DNA to which neither KorA nor KorB is bound. The second term (*Xπ*_X_*k*_A_) represents protein synthesis from DNA to which KorA but not KorB is bound; the protein synthesis rate *k*_A _is scaled by the partial repression parameter *π*_X_. The third term (*Yπ*_Y_*k*_A_) is similar to the second term, but for protein synthesis from DNA to which KorB is bound. No synthesis is possible when both KorA and KorB are bound so there is no term in the equation for this. The fourth term (2*σ*_A_*A*_2_) represents monomerization, in which a single dimer (*A*_2_) dissociates into two monomers. The fifth term (-*λ*_A_*A*_1_^2^) represents dimerization, in which two molecules of KorA bind to form a KorA dimer. The final term (-*γ*_P_*A*_1_) represents protein turnover. Only dilution is taken into account: KorA and KorB are relatively stable proteins and the data modelled come from exponential phase cells, which are growing rapidly. This approximation would not be appropriate if we were modelling stationary phase cells, where the effects of dilution are smaller and so the rate of protein degradation could be more important. Equation 2 describes the rate of change of KorA dimers as a function of time. The terms represent the processes of dimerization, monomerization and protein loss, respectively, and correspond to the equivalent terms in Equation 1. Equations 3 and 4 are similar to Equations 1 and 2; they describe the dynamics for KorB monomers and dimers, respectively.

The ODE model includes a quasi-steady state approximation for the promoter dynamics, arising from the fact that protein associations and dissociations to the DNA strand are faster than protein synthesis and loss. Thus the proportion of unoccupied DNA (*D*) or DNA occupied by KorA (*X*), KorB (*Y*) or KorA and KorB (*Z*) are given by the following hyperbolic terms:(5)(6)(7)(8)(9)(10)(11)(12)

In these equations *k*_1_, *k*_2_, *k*_3_, *k*_4 _are affinities of KorA dimers (*A*_2_) to the DNA strand, KorB dimers (*B*_2_) to the DNA strand, KorA dimers to the DNA-KorB complex and KorB dimers to the DNA-KorA complex, respectively. These hyperbolic terms can be derived from the chemical reaction scheme (see Additional file 1: Chemical reaction scheme) and represent the equilibrium partition over the four possible binding configurations of the DNA. The data to be fitted are the steady state concentrations of KorA and KorB in plasmid RK2 wild type and mutant placed in *E. coli *strains (Table 1).

**Table 1:** Parameters and data in simulations

Parameter/Data	Symbol	Distribution	Value(mode)	cv	Unit
RK2 abundance	*D*_0_	lognormal	4.5	0.5	nM
KorA abundance	A_tot_	lognormal	1600 [18]	0. 5	nM
KorB abundance	B_tot_	lognormal	400 [28]	0. 5	nM
KorB abundance (mutant)	BM_tot_	lognormal	920	0. 5	nM
Scaling parameter for KorA synthesis	*π*_X_	uniform	[0,1]	-	-
Scaling parameter for KorB synthesis	*π*_Y_	uniform	[0,1]	-	-
KorA synthesis	*k*_A_	-	-	-	s^-1^
KorB synthesis	*k*_B_	-	-	-	s^-1^
KorA affinity to DNA	*k*_1_	lognormal	12.9 [18]	0. 5	nM
KorB affinity to DNA	*k*_2_	lognormal	9.3 [22]	0. 5	nM
KorA affinity to KorB-DNA	*k*_3_	lognormal	3.1	0. 5	nM
KorB affinity to KorA-DNA	*k*_4_	lognormal	3.1 [22]	0. 5	nM
KorA monomerization	*σ*_A_	-	-	-	s^-1^
KorB monomerization	*σ*_B_	-	-	-	s^-1^
KorA dimerization	*λ*_A_	lognormal	0.001	0.05	nM^-1^s^-1^
KorB dimerization	*λ*_B_	lognormal	0.001	0.05	nM^-1^s^-1^
Protein degradation	*γ*_P_	lognormal	0.0003875	0.05	s^-1^

cv - coefficient of variation; RK2 abundance (*D*_0_) and protein degradation (*γ*_P_) are calculated for bacteria with the population doubling time of 43 minutes; KorB abundance for plasmid mutant (*BM*_tot_) is unpublished data from C. Thomas et *al*.; affinity of KorA to the KorB-DNA complex (*k*_3_) were assumed to be three fold higher as it is in the case of affinity of KorB to the KorA-DNA complex (*k*_4_); dimerization rates were defined by diffusion properties.

### Two distinct sets of parameter values fit and explain the experimental data

**Figure 2:**
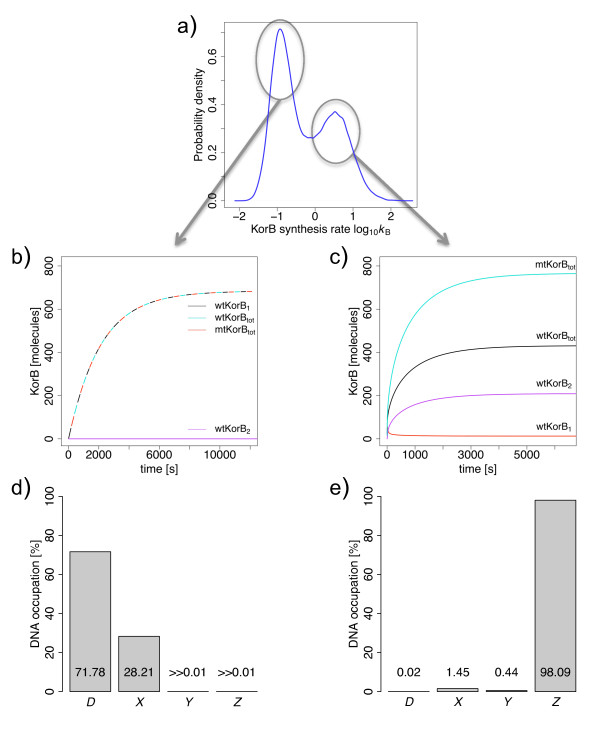
**Two distinct parameter value sets**. (a) Posterior distributions for the protein synthesis rates for KorB and KorA (see Figure. 3) are bimodal. (b) KorB concentrations from model simulations using typical parameter values from the left peak for: dimers in wild type (purple); monomers in wild type (red); total monomers in wild type (black); and total monomers in the plasmid mutant (cyan). The latter three curves are indistinguishable and have been superimposed with dashed lines. For these parameter values, KorB is mainly present as monomers; the same is true for KorA (data not shown). Although the experimentally measured concentration of KorB increased in the mutant strain, the steady state concentrations from these simulations are within the 50% experimental error associated with Western blot analysis. (c) KorB concentrations from model simulations using typical parameter values from the right peak. KorB is mainly present as dimers; the same is true for KorA (data not shown). (d) Proportions of DNA occupation by KorA or KorB dimers in steady state, with left peak parameter values, for empty DNA (D), KorA-DNA complex (X), KorB-DNA complex (Y) and KorA-KorB-DNA complex (Z). The DNA is mostly unoccupied, allowing full transcription to take place, although the presence of a low concentration of KorA dimers indicates some partial repression by KorA. The promoter is very rarely occupied by KorB dimers. (e) Proportions of DNA occupation for right peak parameter values. The promoter is generally repressed, being occupied by both KorA and KorB dimers, and transcription from the unoccupied state is rare.

**Figure 3:**
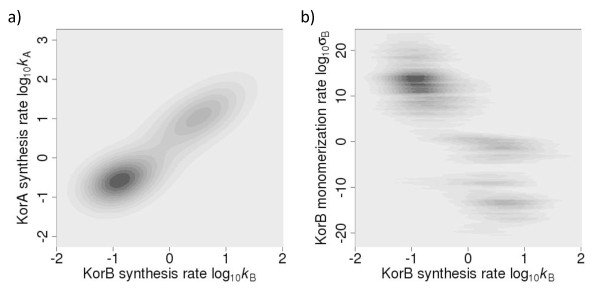
**Posterior distributions of estimated parameters**. (a) Joint posterior distributions of protein synthesis rates (*k_A _*and *k_B_*) in the logarithmic scale. Both parameters are bimodal and the two parameters are positively correlated. (b) Heat map of the joint posterior for the KorB monomerization rate (*σ*_B_) and the KorB synthesis rate (*k*_B_) in the logarithmic scale. The right peak in Figure 2a (high synthesis rate) is associated with low monomerization rate and vice versa.

Parameter estimation has been carried out using the Metropolis-Hastings Monte Carlo scheme as described in the *Methods*. Figure 2a shows marginal posterior distributions of the protein synthesis rate KorB (*k*_B_). The posterior distributions identify two distinct regions from parameter space, each of which can fit the data. The two synthesis rates are themselves highly correlated (Figure 3a) and also correspond to different values of the monomerization rates (Figure 3b). The left-hand peak clearly visible in Figure 2a contains parameter values with low synthesis rates and high monomerization rates. In contrast, the right-hand peak contains parameter values associated with high synthesis rates and low monomerization rates (Figure 3b). Simulations of the model ODEs with typical parameter values associated with the left peak in the marginal posterior distribution (Figure 2a) lead to the conclusion that the Kor proteins present mainly as monomers (Figure 2b), and little repression occurs on the DNA strand (Figure 2d; the unbound D state occurs more than 70% of the time). On the other hand, typical parameter values associated with the right peak result in the Kor proteins being present mainly as dimers (Figure 2c), and the high maximal expression being attenuated by high levels of transcription repression due to cooperative binding of KorA and KorB to the promoter for 98% of the time (Figure 2e, *Z *state). Since the Kor proteins are mostly present as dimers in bacteria [26,18] and the *cco *is mostly repressed [28], the parameters associates with the left peak in the distribution shown in Figure 2a can be ruled out, despite the fact that that peak had a higher posterior probability.

### Monomerization rates are not important and can be excluded

**Figure 4:**
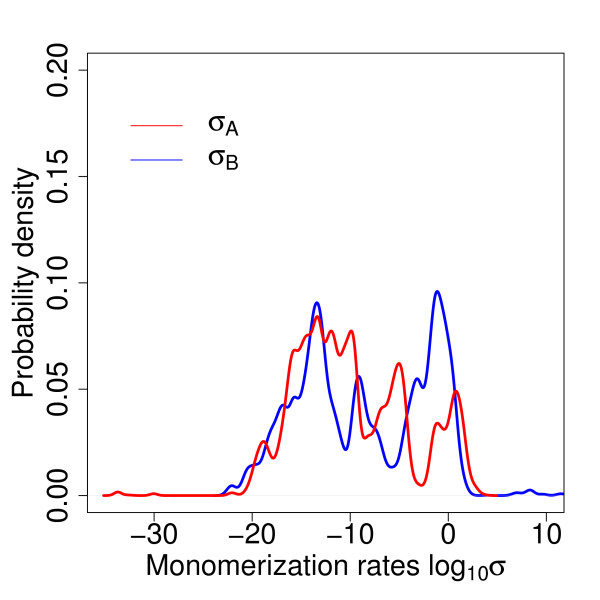
**Posterior distribution of the monomerization rates**. The posterior distribution of the monomerization rates for KorA (blue) and KorB (red) proteins on a logarithmic scale shows the irrelevance of the monomerization rates for the model. The apparently multimodal features of the distributions result from granularity of the sampling over such a wide range; these are artefacts and are not statistically reproducible (data not shown).

Since there was no available experimental knowledge about the monomerization rates these were implemented into the Bayesian inference algorithm with a non-informative prior. After restricting the parameters to the right-hand peak, the marginal posterior distributions for the monomerization rates showed no discernible peak and were spread over a broad range of 40 orders of magnitude (Figure 4).

To establish whether changes in the monomerization rate had any effect on the data fit, ODE calculations were carried out for a number of parameter values while holding other parameters constant. The absolute concentration of KorA and KorB dimers and total concentration varies little (Table 2), and although there is an increase in the number of KorA and KorB monomers, the absolute abundance remains low for all the values examined. On the basis of these analyses, it is reasonable to disregard the monomerization process and set the monomerization rates to 0 for future work since the model fits the data just as well.

**Table 2:** KorA protein abundance for varying monomerization rates

***σ***_**A **_**= *σ***_**B **_**[s**^**-1**^**]**	**A**_**1 **_**[nM]**	**A**_**2 **_**[nM]**	**A**_**tot **_**[nM]**
0	10	838	1686
0.001	19	838	1695
0.01	54	835	1724

*σ*_A_, *σ*_B _- monomerization rates for KorA and KorB, respectively; *A*_1_- KorA monomers, *A*_2 _- KorA dimers, *A*_tot _- KorA total monomers abundance.

### Analyses of joint posteriors is essential for estimation of partial repression parameters

**Figure 5:**
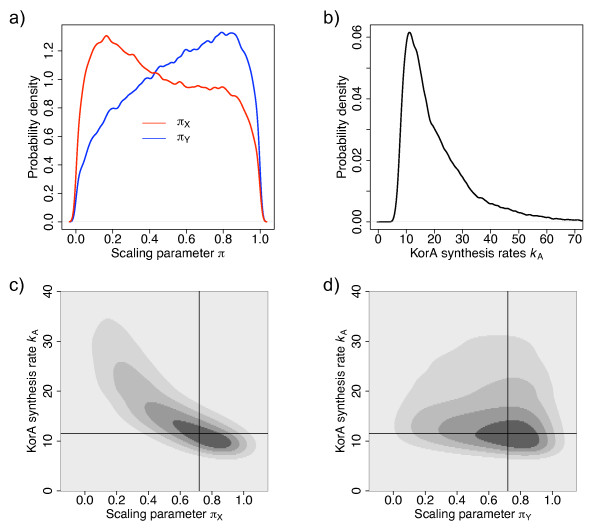
**Posterior distribution of the scaling parameters**. (a) Marginal posterior distributions of the scaling parameters (*π*_X_-blue, *π*_Y _-red) of the protein synthesis rates for KorA-DNA and KorB-DNA complexes, respectively. The distributions do not specify clearly any specific region in the parameter space. (b) Marginal posterior distribution of KorA synthesis rate (*k*_A_). (c) Correlated posterior distributions and selected parameter sets pointed by horizontal and vertical lines for KorA synthesis rate (*k*_A_) and the scaling parameter of the synthesis rate from KorA-DNA complex (*π*_X_) (d) The equivalent plot for KorA synthesis rate (*k*_A_) and the scaling parameter of the synthesis rate from KorB-DNA complex (*π*_Y_).

The parameter estimates were carried out using the marginal posteriors for each parameter. However, our system provided us also with an interesting example where the marginal posteriors were not sufficiently informative and might have even been misleading. The priors of the partial repression scaling parameters were defined by uniform distributions between 0 and 1; the posterior distributions for these parameters are shown in Figure 5a. These posteriors are very broad suggesting that the given parameters are not particularly influential in model fitting. However, they suggest an optimal value for *π*_Y _at about 0.2 and *π*_X _-around 0.8. Figures 5c and 5d show the joint posterior distributions for *π*_X _and *k*_A_, and for *π*_Y _and *k*_A_, respectively. These demonstrate, the latter distribution in particular, that the marginal posterior distributions for *π*_X _and *π*_Y _are biased by the marginal posterior distribution for *k*_A _(Figure 5b). Plots for joint distribution for *π*_*X*_/*π*_*Y *_and *k*_B _look similar (data not shown) because of the high correlation between *k*_A _and *k*_B _(Figure 3a). Better estimations of the partial repression scaling factors can be made by considering these joint distributions. Then, both parameters appear better estimated in the region of 0.72.

### Metabolic Control Analysis reveals complementary important and unimportant parameters

**Figure 6:**
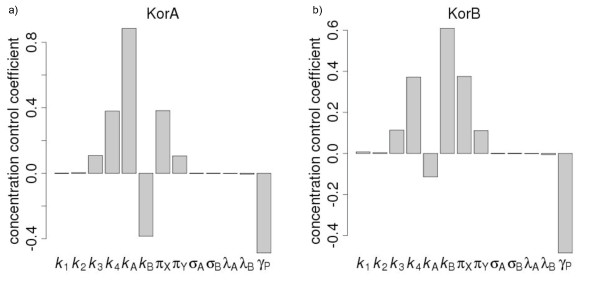
**Concentration Control Coefficients**. Concentration control coefficients for a) KorA and b) KorB shows the sensitivity of the model to each parameter: *k*_A _- KorA synthesis rate, *k*_B _- KorB synthesis rate, *k*_1_-affinity of O_A_1, *k*_2 _- affinity of O_B_1, *k*_3 _- affinity of KorA to KorB-DNA complex, *k*_4 _- affinity of KorB to KorA-DNA complex, σ*_A_*, σ*_B _*- monomerization rates for KorA and KorB, respectively, *λ*_A_, *λ*_B _- dimerization rate for KorA and KorB, respectively, *γ*_P _- protein turnover. The synthesis and turn-over rates are particularly important while the dimerization and monomerization rates are unimportant.

MCA demonstrates that the models are very sensitive to the protein synthesis rates and to protein turnover rates (Figure 6). With regards to protein synthesis rates, for which non-informative priors were used, this result is in line with the posterior distributions from the MCMC, which identified clear peaks for the optimal parameter values. For the protein turnover rates, a highly informative prior was used, since the growth rate had been carefully measured with low error. The posterior distribution obtained closely matched the prior, and thus no further information about the importance of this parameter was obtained using Bayesian inference. MCA was required to highlight the sensitivity of the model of this parameter.

The protein synthesis rates show positive control coefficients for the abundance of the associated protein, and a negatively control coefficient for the abundance of the alternate protein. There is also moderate control of the protein abundances by the cooperative affinity of KorA and KorB to the DNA (*k*_3_, *k*_4_). This results from the occupation of this state for the greatest proportion of time (Figure 2e). The other affinities are less important because the DNA is only in unoccupied or partly occupied states for a small proportion of time. MCA also revealed the low impact of the monomerization rates on the model (Figure 6). This is in agreement with the results obtained with the MCMC-based approach and thus provides further justification for the exclusion of these parameters in future work.

### Partial repression models give very different protein synthesis rates and suggest model elimination

In the final analysis we made further inferences about the extent of partial repression of the *cco *by KorA and KorB dimers. We utilised the experimental measurement that level of repression associated with the presence of KorA and KorB at physiological levels relative to their absence is 91.8-fold [28]. To do this, we refined the main model described above to take into consideration different possible mechanisms of partial repression. Five competing models were set up, using the same equations as above, but in which some parameter values were fixed at 0 or 1. Specifically, protein expression while either KorA or KorB are bound were considered either as none (*π*_X _= *π*_Y _= 0), full (*π*_X _= *π*_Y _= 1) or partial (*π*_X _and *π*_Y _between 0 and 1) (Table 3). In all cases, the monomerization rate was set to zero. For each model, the protein synthesis rate was estimated using an appropriately modified MCMC scheme. All models fit the protein expression data equally well (see Additional file 2: Protein abundance obtained for five competing models). Because the monomerization rates were set to zero, the posterior distributions for the protein synthesis rates had only one peak (data not shown); the estimates from these distributions are given in Table 3. It is important to remember that the estimated protein synthesis rates always define the maximum rate of protein synthesis, namely, when expression from the empty DNA strand takes place, and not the net rate once repression is factored in.

**Table 3:** Estimated protein synthesis rates and their adequate scaling parameters for five different models

Model	***k***_**A **_**[s**^**-1**^**]**		***π***_**X**_		***k***_**B **_**[s**^**-1**^**]**		***π***_**Y**_	
	m	cv	m	cv	m	cv	m	cv
11	7.9	0.2	-	-	2.3	0.2	-	-
uu	11.5	1.4	0.72	0.5	3.2	1.3	0.72	0.4
u0	14.0	2.4	0.75	0.45	4.0	2.5	-	-
0u	43.0	1.5	-	-	11.6	1.6	0.8	0.4
00	735.0	0.3	-	-	231.0	0.2	-	-

*k*_A_, *k*_B _- protein synthesis rate for KorA and KorB, respectively; *π*_X_,*π*_Y _- scaling parameter for KorA-DNA and KorB-DNA complexes respectively; m - median; cv - coefficient of variation; models: the first and second signs stand for expression from complexes when KorA or KorB are bound to the DNA, respectively, 1- no repression, u - partial repression, 0 - total repression

**Figure 7:**
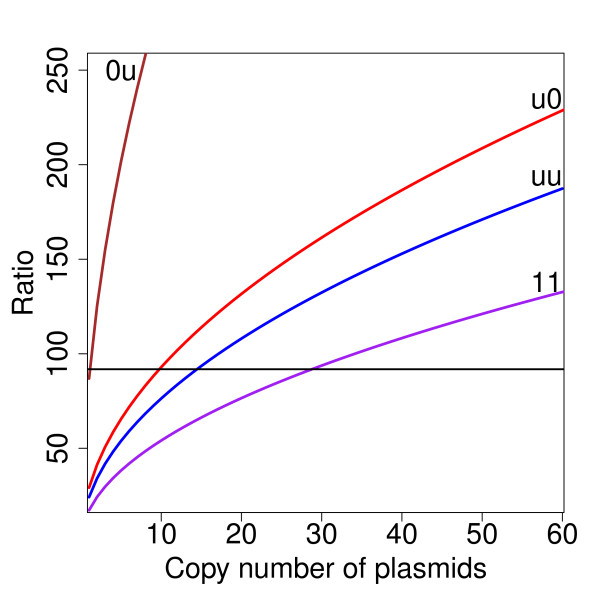
**Dependence of the unrepressed to repressed ratio on the copy number of plasmids**. Models: brown - 0u, red - u0, blue - uu, purple - 11. The reported 91.8-fold repression ratio is shown as a horizontal line. The ratios for the u0 and uu models cross this line at a realistic plasmid copy number. The 0u model crosses the line at an unrealistically low plasmid copy number, and the 00 model has much higher ratios (data not shown). The 11 model crosses the line at an unrealistically high plasmid copy number; models: the first and second signs stand for expression from complexes when KorA or KorB are bound to the DNA, respectively, 1 - no repression, u - partial repression, 0 - total repression.

Each of these models gives a very different maximal rate of synthesis, ranging over two orders of magnitude, which should lead to quite different protein abundances if no repressors are present (Table 3). This suggests that a possible way to differentiate between the models is by measurement of protein abundance when the promoter is unrepressed, i.e. when neither KorA nor KorB are able to bind to the DNA. The differences between the five models can also be expressed as the ratio between the protein abundance in the disrupted and the wild type systems (Table 4). The closest of our model predictions to the 91.8-fold repression were the u0 model, with 99-fold depression and the uu model, with 81-fold repression, in the 11-plasmid model (Table 4). Having included the reported normalized plasmid copy numbers into the model to more closely match the experimental results reported in [28] (0.41 for pDM3.1 reporter gene and 1.59 for pRK24 plasmid), we estimated 89-fold repression for the uu model and 120-fold repression for the u0 model. Although this appeared to favour the uu model, taking into account the experimental error, we considered both regulation scenarios as plausible. The model u0 predicts maximal rate of KorA and KorB production of 14 s^-1 ^and 4 s^-1^, 75%-maximal expression when KorA is bound to the DNA, and total repression when KorB is bound (Table 3). The uu model is also compatible with the data with slightly lower maximal rates of KorA and KorB production, and 72%-maximal production when either KorA or KorB dimers alone are bound to the DNA. The 11 model, in which neither KorA nor KorB alone have any repressive effect, requires a plasmid copy number of 29. This is unlikely to be biologically plausible in the experiment with low plasmid copy number. Importantly, we can exclude models where there is no expression when KorA is bound to the DNA - the 00 and 0u models (Figure 7) - as they are not compatible with the experimental data. The ratios for these models are either too high, even for a plasmid copy number of 1 (ratio = 1058; model 00, data out of the plot range in the Figure 7), or for the 0u model - requiring an unrealistic copy number of 1-2 plasmids per cell to obtain 91.8-fold repression.

**Table 4:** Ratio of protein abundance in the model without repression to the model under consideration

Model			11 plasmids	
	***k***_**A **_**[s**^**-1**^**]**	**A**_**tot**_ [nM]	**A**_**tot **_**(no repression) [nM]**	Ratio
11	7.9	1576	91741	58
uu	11.5	1649	133548	81
u0	14	1649	162582	99
0u	43	1627	499355	307
00	735	1649	133548	5308

Ratios for the system with 11 plasmids; *k*_A_, *k*_B _- protein synthesis rate for KorA and KorB, respectively; *A*_tot_, *B*_tot _- total monomer abundance of KorA and KorB, respectively; Ratio A, Ratio B - a ratio of protein abundance between the model with no repression and the system under consideration for KorA and KorB, respectively; models: the first and second signs stand for expression from complexes when KorA or KorB are bound to the DNA, respectively, 1 - no repression, u - partial repression, 0 - total repression.

## Discussion

In this work, we have developed a mathematical model for the regulation of the global regulators KorA and KorB of RK2 plasmids, a natural example of an autogenous, negatively self-regulated transcriptional system. The combination of Bayesian inference using MCMC with dynamical models has allowed us to integrate mechanisms and data in a systems biology context, and enabled us to explore hypotheses about unknown parameters and mechanisms.

One great advantage of the Metropolis-Hastings algorithm is that, if used carefully, it can reveal multimodal distributions of parameter values providing good fit to data, which can be thought of as 'local optima' within classical parameter estimation such as least squares. In our case, these had different biological interpretations. Although we could rule out one set of parameter values based on the knowledge that KorA and KorB are mostly present as dimers, it is easy to envisage applications where such relevant information would not be available. Had that been the case, the methodology would have highlighted a crucial experiment to carry out.

The MCMC approach can also assess the sensitivity of the model to parameter estimates using the spread of the posterior distributions. We have found that our model is sensitive to the rates of protein synthesis, and insensitive to the rates of monomerization. However, there are two limitations. First, MCMC does not indicate the direction of parameter changes. Second, where tight prior information has been provided, such as in our case for the protein turnover rates, we were unable to glean further information.

These limitations have been overcome by the complementary use of MCA for parameter sensitivity analysis, which has provided us with quantitative and qualitative information about parameters. MCA was able to confirm the importance of the protein synthesis rates and the irrelevance of the monomerization rates. In addition, MCA identified negative correlation between the protein synthesis rates for KorA/KorB and the concentration of KorB/KorA, respectively. Finally, MCA revealed the sensitivity of the system to the protein turnover rates, highlighting the need for careful measurement of these parameters. In this model, the protein turnover parameters represent the process of dilution due to cell growth. Since the carriage of plasmids has a potential impact on host cell growth rate, this would suggest that the plasmid system itself is highly sensitive to that impact, and could imply that there would be strong evolutionary pressure to reduce the impact.

With statistical modelling, the identification of correlated parameters can indicate a need to reformulate the model. In this work, the identification of correlations in the joint posterior distributions for the rate of protein synthesis and degree of partial repression raises the question as to whether the model is best specified as described, or whether an alternative formulation might be more suited for inference in which the posterior distributions for the parameters would not be correlated. Two alternative model formulations could be considered. The first formulation would be to specify different parameters for the protein synthesis of KorA and KorB in each of the partial repression scenarios, with appropriate joint priors to ensure that KorA and KorB act as repressors in the model. However, this formulation has two problems. The first is that the resulting model would not be biologically realistic: *korA *and *korB *are transcribed together on the same operon, and then translated separately. Thus partial repression has the same impact on both genes, which is captured in the formulation used, but not in this alternative. From that perspective, it perhaps not surprising that the correlation emerges, as the synthesis of the two proteins are (biologically) correlated through joint transcription. The second problem is that the model would have 6 parameters instead of 4, which would significantly reduce the potential for successful parameter estimation. The second alternative formulation would be to build a model that explicitly includes separate equations for transcription of the *korABF *operon, and translation of each of the proteins. However, such a model would also have many more parameters than the one used. In the absence of experimental data of mRNA abundance and turnover, it would be impossible to infer the unknown parameters. Taking these aspects of alternative model formulations into account, we conclude that our original model is likely to be optimal: it combines biological realism with the capacity to infer parameters for which there is no prior knowledge.

In this model, the interactions between KorA and KorB dimers take place only on the DNA strand. It has been suggested that KorA and KorB dimers interact in solution (David Scott, personal communication). However, since we predict that the DNA is bound by both proteins 98% of the time (Figure 2e), we expect that such potential interactions should not affect the results.

The analysis of various model scenarios representing different possible mechanisms of partial repression have revealed that partial repression by KorA dimers is more compatible with the data than complete repression by KorA. This result appears surprising as KorA binds in the -10 region of the promoter, precisely overlapping the RNAP binding site [18]. While RNAP is necessary to open the DNA strand for transcription initiation, one might expect complete repression by KorA. It is important to note, however, that the model does not explicitly include competition between KorA and RNA polymerase at their overlapping binding sites. As a consequence, the prediction of partial repression by KorA can be explained as an indication that the competition between KorA and RNA polymerase is relevant for limiting transcription.

The simulations used for the model comparison shown in Figure 7 were carried out using point estimates of the model parameters chosen from the posterior distributions for each of the models. An alternative approach is to resample from the posterior distributions to obtain a statistical ensemble of model parameters and base model comparisons on that ensemble. The results of that approach (Additional File 3) lead to the same conclusions: the models that include partial repression by KorA are most compatible with the repression ratio data.

## Conclusions

We have devised a mathematical model for the transcription regulation of the central control operon of RK2 plasmids by the global regulators KorA and KorB. By using an approach that couples mechanistic dynamical models with Bayesian inference, we have answered the five specific research questions we posed. First, the experimental data available for the cco system are compatible with our knowledge about its regulatory mechanisms. Second, they enable us to estimate unknown parameters such as protein synthesis and monomerization rates. Moreover, two distinct sets of parameter values can explain the experimental data, highlighting the importance of measuring the relative abundance of dimers to monomers. Third, the monomerization rate is not particularly relevant to the model formulation and can thus be neglected; and estimation of partial repression is dependent on the estimation of the protein synthesis rates and thus these parameters cannot be estimated independently. By using MCA we have also revealed the sensitivity of the model to the protein turn-over rates. Fourth, total repression by KorB alone is incompatible with the de-repression data, and it is likely that competition between KorA and RNA polymerase is an important factor in this particular regulatory system. Fifth, and finally, we have shown that MCMC and MCA are complementary approaches for parameter sensitivity analysis for our model. In summary, we highlight the potential of combining dynamical modelling, statistical inference and sensitivity analyses for deepening our understanding of gene regulatory systems and exploring biological hypotheses about their mechanisms of action.

## Methods

### Distributions for protein expression data

The phenotyopic read-outs are the total protein monomer abundance of KorA (*A*_tot_), KorB (*B*_tot_) and KorB mutant KorB (*BM*_tot_) (cooperativity between KorA and KorB knock out); these have been measured experimentally [28; unpublished data from Thomas et *al*.]. Distributions for the data are defined by lognormal distributions with mode equal to measured value and a coefficient of variation equal to 50%, reflecting the level of variability observed experimentally (Table 1). For the simulations used to estimate the scaling parameters, the coefficient of variation for data and the measured parameters was reduced to 10% to aid convergence. Lognormal distributions are chosen because the experimental error is expressed as a percentage of measured abundance. Protein abundances were measured as the numbers of total monomers per cell based on total monomers measured for a known number of bacteria. Molar concentrations of proteins were calculated for the estimated cell volume in the appropriate growth phase, 4.15 μm^3 ^for E.coli [28].

### Distributions for model parameters

The affinities of KorA to the DNA strand (*k*_1_), KorB to the DNA strand (*k*_2_), KorA to the KorB-DNA complex (*k*_3_), KorB to the KorA-DNA complex (*k*_4_) and also DNA abundance (*D*_0_) have also been measured experimentally [18,22], and their prior distributions are also lognormal (Table 1). The affinities of KorA and KorB to the complexes were assumed to be equal to the affinity of KorB to the IncC1-DNA complex at O_B_1 operator since IncC1 increases the affinity binding factor to 3.2 [22] when KorA increases the binding factor to about 3.4 [16]. Other parameters such as the dimerization rates were defined by diffusion properties (*λ*_A_, *λ*_B_) and the protein degradation rate (*γ*_P_) with the coefficient of variation equal to 5%. The protein degradation rate *γ*_P_, which is based only on dilution due to the fact that KorA and KorB are relatively stable proteins, was estimated from bacteria population doubling time of around 43 min. The protein synthesis and monomerization rates were entirely unknown and foe this reason, non-informative uniform priors were used for these parameters. Priors for the scaling parameters *π*_X_, *π*_Y _were defined by uniform distributions between 0 and 1.

### Monte Carlo Simulations

Parameter estimations were carried out using the Metropolis-Hastings algorithm [29] coupled with the steady state of the dynamical model described in the *Results*. For a selection of the next set of the rate parameters, lognormal proposal distributions were used based on logarithm of random values selected from a normal distribution with mean 0 and standard deviation 0.05; this can be achieved either by adding a normal deviate to the log of the parameter, or by multiplying the parameter by the exponential of the normal deviate. Lognormal proposals are chosen because: (i) they ensure parameters remain positive; (ii) we have no knowledge of the scale of the parameters with un-informative priors and lognormal proposals allow searching over all orders of magnitude in an unbiased way; and (iii) for parameters with informative priors the experimental errors are expressed as percentage of mean and therefore lognormal distributions are more natural. For the two scaling parameters with uniform bounds and priors, a normal proposal distribution with mean 0 and standard deviation 0.08 was used. The variances of these distributions were empirically chosen to ensure acceptance probabilities close to 0.234 [38]. The parameter set was divided into three blocks: parameters defined by lognormal prior distributions and two separate blocks with a parameter defined by uniform prior distribution. Every iteration parameters only from one block were updated while other parameters remained unchanged. The systems did not require any additional searching techniques since satisfactory convergence was achieved. Simulations that consisted of 4,000,000 iterations were long enough to ensure repeatability.

### Likelihood calculations

The joint likelihood function was given by the product of three terms, one for each data point, consisting of the probability density for the steady state concentrations of KorA and KorB in the wild-type model, and KorB in the mutant model, under lognormal distributions centred on the measured data. Calculations of the steady states of the system used multidimensional root finding were carried out using the discrete Newton algorithm of the GSL library [39] encoded in C++. For the same set of parameters, the root finding calculations were carried separately for wild type and mutant models. The only differences between these models were the values of the parameters describing protein affinities of cooperative DNA binding - the affinity of either KorA or KorB to DNA-KorB or DNA-KorA complexes, respectively. These affinities were set to the same values as the affinities of KorA or KorB binding to the naked DNA strand (k_3 _= k_1 _and k_4 _= k_2_).

### Calculations using dynamical simulations

Additionally, for model simulations with specific parameter value sets (data presented in Table 2, Table 4, Additional file 2, Figure 2b, c, d, e, and Figure 6), ODE calculations were run in C++ using the cvode solver with Newton iterations provided by the Sundials library [40]. Moreover, for quantitative sensitivity analyses, the MCA [36,37] were carried out by direct calculations of the concentration control coefficient from the calculations using ODE simulations.

## Competing interests

The authors declare that they have no competing interests.

## Authors' contributions

DH carried out all simulations and wrote the manuscript draft. CMT participated in the design of the study and helped write the manuscript. DJS devised the study, devised the initial model and helped write the manuscript. All authors read and approved the final manuscript.

## Supplementary Material

Additional file 1 **Chemical reaction scheme**. A chemical reaction scheme from which the model has been derived. a) association/disosiation of KorA or KorB dimers (*A*_2_, *B*_2_) to/from the empty DNA strand (*D*), KorA-DNA complex (*X*), KorB-DNA complex (*Y*), KorA-KorB-DNA complex (*Z*), *k*_on _- association rate, *k*_off1_, *k*_off2_, *k*_off3_, *k*_off4 _- protein dissociation rates; b) and c) KorA or KorB monomers production (*A*_1_, *B*_1_) from empty DNA strand (*D*) with maximum synthesis rates *k*_A _and *k*_B _for KorA and KorB, respectively, from KorA-DNA (*X*) and KorB-DNA complexes (*Y*) with scaled protein synthesis rates by *π*_X _and *π*_Y_, respectively, due to partial repression; d) dimerizations (*λ*_A_, *λ*_B_) and monomerizations (*σ*_A_, *σ*_B _) of KorA and KorB; e) KorA and KorB, monomers and dimers (*A*_1_, *B*_1_, *A*_2_, *B*_2_) dilution with a rate γ*_P_*. Click here for file

Additional file 2 **Protein abundance obtained for five competing models**. Table legend: Protein abundance obtained with ODE calculations for each model with responding parameters, which were estimated with Bayesian inference, and data for reference; *k*_A_, *k*_B _- protein synthesis rates for KorA and KorB, respectively; *π*_X_,*π*_Y _- scaling parameter for KorA-DNA and KorB_DNA complexes, respectively; KorA_tot_, KorB_tot_, KorBM_tot _- total monomers abundance of KorA, KorB in the wild type and KorB in the mutant; models: the first and second signs stand for expression from complexes when KorA or KorB are bound to the DNA, respectively, 1 - no repression, u - partial repression, 0 - total repression. Click here for file

Additional File 3 **Ensemble comparison of competing models**. Models: brown - 0u, red - u0, blue - uu, purple - 11. The reported 91.8-fold repression ratio is shown as a horizontal line. The calculations of repression indexes (ratios) from the re-sampled posterior distributions using every 360th sample entry as uncorrelated samples (over 11000 samples for each model). For each sample we calculated ratio of KorA total monomers abundance unregulated to regulated system. The calculations were run for each model for plasmid copy numbers from 1 to 60. The solid coloured lines represent the values indicated by a mode of the log normal posterior distribution fitted to each model and plasmid copy number. The standard errors are to small to be distinguished on the plot: they vary between 0.007 and 0.070 for the 0u model, 0.004 an 0.040 for the u0 model, 0.002 and 0.016 for the uu model, 0.0001 and 0.0012 for the 11 model. The ratios for the u0 and uu models cross this line a little lower than the attested plasmid copy number (~11), with values of 5 and 7 respectively. The 0u model crosses the line at an unrealistically low plasmid copy number, and the 00 model has much higher ratios (data not shown). The 11 model crosses the line at an unrealistically high plasmid copy number. Model nomenclature: the first and second symbols stand for expression from complexes when KorA or KorB are bound to the DNA, respectively, 1 - no repression, u - partial repression, 0 - total repression. Click here for file
