# Supplementary figures and images for: Global transcription regulation of RK2 plasmids: a case study in the combined use of dynamical mathematical models and statistical inference for integration of experimental data and hypothesis exploration
Source: BMC Syst Biol. 2011 Jul 29;5:119. doi: 10.1186/1752-0509-5-119 (PMC3199767; doi:10.1186/1752-0509-5-119)

a)

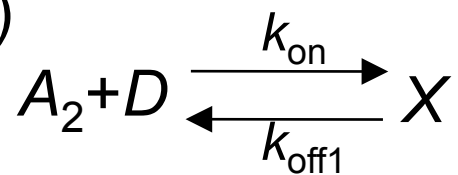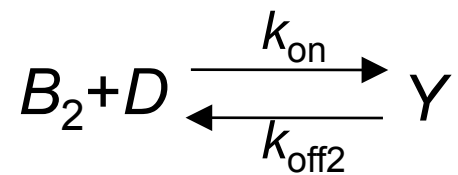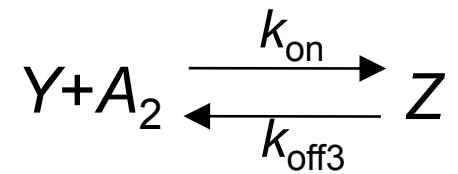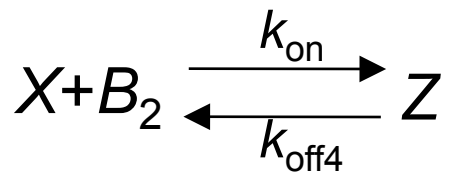

b)

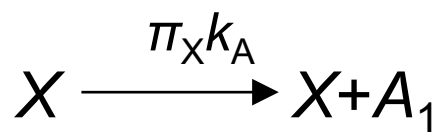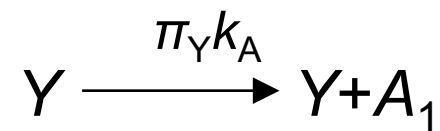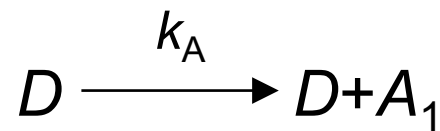

c)

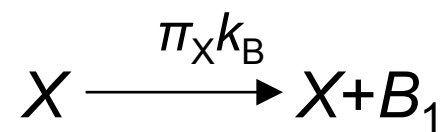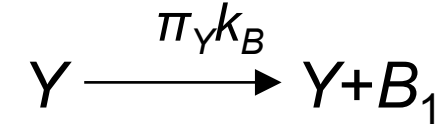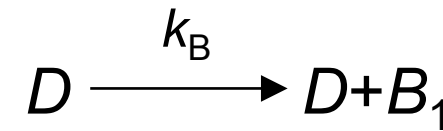

d)

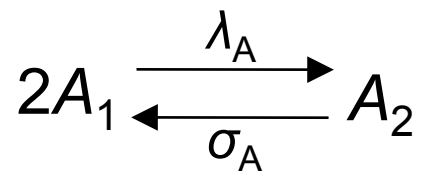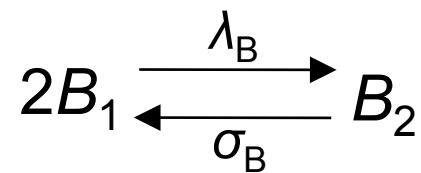

e)

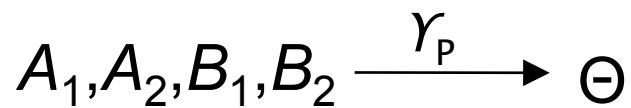

Supplement: Additional file 1 — Chemical reaction scheme. A chemical reaction scheme from which the model has been derived. a) association/disosiation of KorA or KorB dimers (A2, B2) to/from the empty DNA strand (D), KorA-DNA complex (X), KorB-DNA complex (Y), KorA-KorB-DNA complex (Z), kon - association rate, koff1, koff2, koff3, koff4 - protein dissociation rates; b) and c) KorA or KorB monomers production (A1, B1) from empty DNA strand (D) with maximum synthesis rates kA and kB for KorA and KorB, respectively, from KorA-DNA (X) and KorB-DNA complexes (Y) with scaled protein synthesis rates by πX and πY, respectively, due to partial repression; d) dimerizations (λA, λB) and monomerizations (σA, σB ) of KorA and KorB; e) KorA and KorB, monomers and dimers (A1, B1, A2, B2) dilution with a rate γP. [file 1752-0509-5-119-S1.PDF]
