# Supplementary material for: Global transcription regulation of RK2 plasmids: a case study in the combined use of dynamical mathematical models and statistical inference for integration of experimental data and hypothesis exploration
Source: BMC Syst Biol. 2011 Jul 29;5:119. doi: 10.1186/1752-0509-5-119 (PMC3199767; doi:10.1186/1752-0509-5-119)
Supplement: Additional file 2 — Protein abundance obtained for five competing models. Table legend: Protein abundance obtained with ODE calculations for each model with responding parameters, which were estimated with Bayesian inference, and data for reference; kA, kB - protein synthesis rates for KorA and KorB, respectively; πX,πY - scaling parameter for KorA-DNA and KorB_DNA complexes, respectively; KorAtot, KorBtot, KorBMtot - total monomers abundance of KorA, KorB in the wild type and KorB in the mutant; models: the first and second signs stand for expression from complexes when KorA or KorB are bound to the DNA, respectively, 1 - no repression, u - partial repression, 0 - total repression. [file 1752-0509-5-119-S2.DOC]

### Additional file 2 – Protein abundance obtained for five competing models

| Model | *k*_A_ [s^-1^] | *π*_X_ | *k*_B_ [s^-1^] | *π*_Y_ | KorA_tot_ | KorB_tot_ | KorBM_tot_ |
| --- | --- | --- | --- | --- | --- | --- | --- |
| 11 | 7.9 | 1.00 | 2.3 | 1.00 | 1598 | 468 | 834 |
| uu | 11.5 | 0.72 | 3.2 | 0.72 | 1669 | 467 | 830 |
| u0 | 14.0 | 0.75 | 4.0 | 0 | 1649 | 469 | 805 |
| 0u | 43.0 | 0 | 11.6 | 0.80 | 1629 | 453 | 882 |
| 00 | 735.0 | 0 | 231.0 | 0 | 1607 | 503 | 739 |
| Data | - | - | - | - | 1600 | 400 | 920 |

Protein abundance obtained with ODE calculations for each model with responding parameters, which were estimated with Bayesian inference, and data for reference; *k*_A_, *k*_B_ – protein synthesis rates for KorA and KorB, respectively; *π*_X_,*π*_Y_ – scaling parameter for KorA-DNA and KorB_DNA complexes, respectively; KorA_tot_, KorB_tot_, KorBM_tot_ – total monomers abundance of KorA, KorB in the wild type and KorB in the mutant; models: the first and second signs stand for expression from complexes when KorA or KorB are bound to the DNA, respectively, 1 – no repression, u – partial repression, 0 – total repression
