# Supplementary material for: Global transcription regulation of RK2 plasmids: a case study in the combined use of dynamical mathematical models and statistical inference for integration of experimental data and hypothesis exploration
Source: BMC Syst Biol. 2011 Jul 29;5:119. doi: 10.1186/1752-0509-5-119 (PMC3199767; doi:10.1186/1752-0509-5-119)
Supplement: Additional File 3 — Ensemble comparison of competing models. Models: brown - 0u, red - u0, blue - uu, purple - 11. The reported 91.8-fold repression ratio is shown as a horizontal line. The calculations of repression indexes (ratios) from the re-sampled posterior distributions using every 360th sample entry as uncorrelated samples (over 11000 samples for each model). For each sample we calculated ratio of KorA total monomers abundance unregulated to regulated system. The calculations were run for each model for plasmid copy numbers from 1 to 60. The solid coloured lines represent the values indicated by a mode of the log normal posterior distribution fitted to each model and plasmid copy number. The standard errors are to small to be distinguished on the plot: they vary between 0.007 and 0.070 for the 0u model, 0.004 an 0.040 for the u0 model, 0.002 and 0.016 for the uu model, 0.0001 and 0.0012 for the 11 model. The ratios for the u0 and uu models cross this line a little lower than the attested plasmid copy number (~11), with values of 5 and 7 respectively. The 0u model crosses the line at an unrealistically low plasmid copy number, and the 00 model has much higher ratios (data not shown). The 11 model crosses the line at an unrealistically high plasmid copy number. Model nomenclature: the first and second symbols stand for expression from complexes when KorA or KorB are bound to the DNA, respectively, 1 - no repression, u - partial repression, 0 - total repression. [file 1752-0509-5-119-S3.DOC]

**Dorota Herman, Chris Thomas and Dov Stekel Additional File 3**

**Ensemble comparison of competing models**


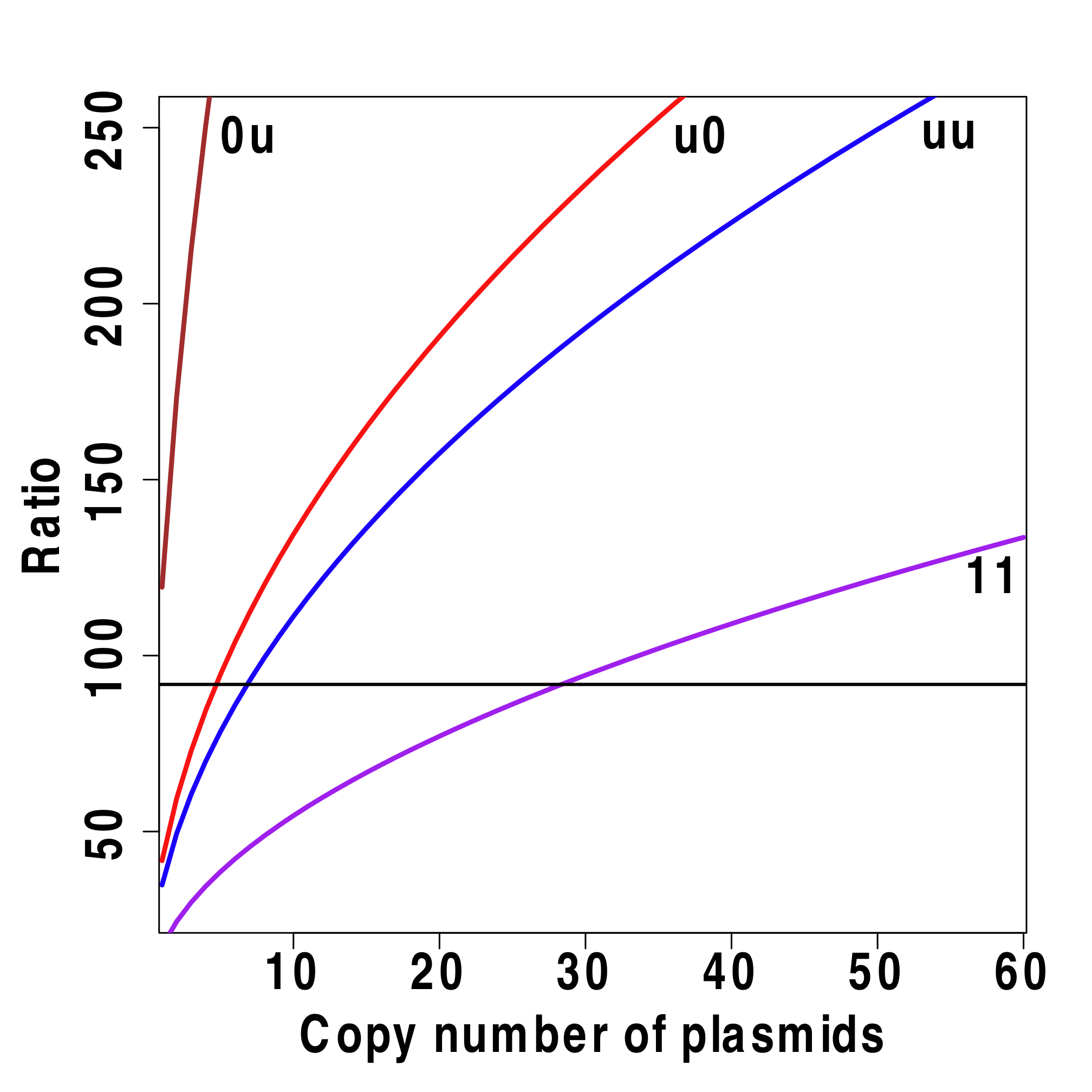


Models: brown – 0u, red – u0, blue – uu, purple – 11. The reported 91.8-fold repression ratio is shown as a horizontal line. The calculations of repression indexes (ratios) from the re-sampled posterior distributions using every 360th sample entry as uncorrelated samples (over 11000 samples for each model). For each sample we calculated ratio of KorA total monomers abundance unregulated to regulated system. The calculations were run for each model for plasmid copy numbers from 1 to 60.

The solid coloured lines represent the values indicated by a mode of the log normal posterior distribution fitted to each model and plasmid copy number. The standard errors are to small to be distinguished on the plot: they vary between 0.007 and 0.070 for the 0u model, 0.004 an 0.040 for the u0 model, 0.002 and 0.016 for the uu model, 0.0001 and 0.0012 for the 11 model. The ratios for the u0 and uu models cross this line a little lower than the attested plasmid copy number (~11), with values of 5 and 7 respectively. The 0u model crosses the line at an unrealistically low plasmid copy number, and the 00 model has much higher ratios (data not shown). The 11 model crosses the line at an unrealistically high plasmid copy number.

Model nomenclature: the first and second symbols stand for expression from complexes when KorA or KorB are bound to the DNA, respectively, 1 – no repression, u – partial repression, 0 – total repression.
